# Optical Coherence Tomography Angiography in Neurodegenerative Disorders †

**DOI:** 10.3390/jcm9061706

**Published:** 2020-06-02

**Authors:** Marco Pellegrini, Aldo Vagge, Lorenzo Ferro Desideri, Federico Bernabei, Giacinto Triolo, Rodolfo Mastropasqua, Chiara Del Noce, Enrico Borrelli, Riccardo Sacconi, Claudio Iovino, Antonio Di Zazzo, Matteo Forlini, Giuseppe Giannaccare

**Affiliations:** 1 Ophthalmology Unit, S. Orsola-Malpighi University Hospital, University of Bologna, 40138 Bologna, Italy; marco.pellegrini@hotmail.it (M.P.); federico.bernabei89@gmail.com (F.B.); 2 University Eye Clinic, DINOGMI, Polyclinic Hospital San Martino IRCCS, 16132 Genoa, Italy; lorenzoferrodes@gmail.com (L.F.D.); delnocechiara@gmail.com (C.D.N.); 3 Ophthalmology Department, Fatebenefratelli and Ophthalmic Hospital, ASST-Fatebenefratelli-Sacco, 63631 Milan, Italy; giacinto.triolo@gmail.com; 4 Institute of Ophthalmology, University of Modena and Reggio Emilia, 41121 Modena, Italy; rodolfo.mastropasqua@gmail.com; 5 Department of Ophthalmology, Hospital San Raffaele, University Vita Salute San Raffaele, 20132 Milan, Italy; borrelli.enrico@yahoo.com (E.B.); ric.sacconi@gmail.com (R.S.); 6 Department of Surgical Sciences, Eye Clinic, University of Cagliari, 09124 Cagliari, Italy; claudioiovino88@gmail.com; 7 Department of Ophthalmology, University Campus Bio-Medico of Rome, 00128 Rome, Italy; antoniodizazzo@gmail.com; 8 Domus Nova Hospital, 48121 Ravenna, Italy; matteoforlini@gmail.com; 9 Department of Ophthalmology, Ospedale dello Stato della Repubblica di San Marino, 47893 Città di San Marino, San Marino; 10 Department of Ophthalmology, University “Magna Graecia”, 88100 Catanzaro, Italy; giuseppe.giannaccare@gmail.com

**Keywords:** optical coherence tomography angiography, neurodegenerative disorders, Alzheimer’s disease, Parkinson’s disease, multiple sclerosis, glaucoma

## Abstract

Retinal microcirculation shares similar features with cerebral small blood vessels. Thus, the retina may be considered an accessible ‘window’ to detect the microvascular damage occurring in the setting of neurodegenerative disorders. Optical coherence tomography angiography (OCT-A) is a non-invasive imaging modality providing depth resolved images of blood flow in the retina, choroid, and optic nerve. In this review, we summarize the current literature on the application of OCT-A in glaucoma and central nervous system conditions such as Alzheimer’s disease, Parkinson’s disease, and multiple sclerosis. Future directions aiming at evaluating whether OCT-A can be an additional biomarker for the early diagnosis and monitoring of neurodegenerative disorders are also discussed.

## 1. Introduction

### 1.1. Retinal and Ccerebral Microvasculature

The retina and optic nerve are considered part of the central nervous system. During embryogenesis, they originate as outgrowths of the of the developing brain, specifically the embryonic diencephalon [1]. Anatomically, the retina contains glial cells as well as interconnected neurons composed by a cell body, dendrites, and an axon, resembling the cellular architecture of the cerebral cortex. The visual signal originates from the photoreceptors and reaches the retinal ganglion cells through various intermediate neuronal cells, including the bipolar, amacrine, and horizontal cells. The axons of retinal ganglion cells join to form the optic nerve, which is surrounded by all three meningeal layers and is covered by myelin produced by oligodendrocytes, similarly to all central nervous system fiber tracts [2].

In terms of the microvasculature, retinal and cerebral small blood vessels share many common features. Both cerebral and retinal microcirculation are high oxygen extraction systems whose flow is dependent on local neuronal activity [3]. In addition, they have mechanisms of autoregulation to maintain a relatively constant blood flow despite variation in perfusion pressure [4,5]. Finally, the cerebral and retinal neural environments are protected from blood-borne compounds and pathogens by specialized barriers. The inner blood-retinal barrier comprises tight junctional complexes between nonfenestrated endothelial cells surrounded by astrocytic end feet, and thus closely resembles the blood-brain barrier [6].

Given the strong anatomical and physiological homologies between these two tissues, it has been suggested that central nervous system disorders may be associated with distinctive retinal changes. Recent advances in retinal imaging allow the non-invasive visualization of retinal components at a cellular level. In this perspective, the retina may represent an easy accessible ‘window’ to evaluate the cerebral neuronal and microvascular damage [7,8,9].

### 1.2. Retinal Ganglion Cells

Among at least 30 types of RGCs isolated in the human retina, only few of them seem to play a key role for the visual function [10]. The P-pathway includes very small midget cells (or P-cells) that are connected to single-cone midget bipolar cells in the central 2 mm around the fovea and project to the parvocellular layers of the lateral geniculate nucleus [11]. P-cells have small cell bodies, thin axons, and narrow dendritic trees with a more bushy and dense branching pattern [12]. Conversely, the parasol cells (or M-cells) constitute a smaller proportion of RGCs, are characterized by large cell bodies, thick axons, and wide radial branching dendritic trees and project to the magnocellular layers of the lateral geniculate nucleus. Both RGC classes increase in size with increasing distance from the foveal slope, maintaining their distinct branching pattern at all eccentricities, but the average P-cell dendritic-field diameter is smaller than mean M-cell dendritic field throughout the retina.

In glaucoma, the pattern of RGC loss in glaucoma has been thoroughly investigated by several OCT studies. It is well established that initially the peripapillary retinal nerve fiber layer (RNFL) is mainly affected in the inferior and superior quadrants [13,14], whereas macular ganglion cell-inner plexiform layer (GCIPL) defects are preferentially located at the inferior-temporal and superior-temporal macular sectors [15,16]. This clearly points to a predominant damage of the inferior and superior optic nerve head quadrants where M-cells are preferentially located, with a relative sparing of the temporal sector (P-cells) [17]. In Alzheimer’s disease (AD), RNFL thinning appears more pronounced in the superior and inferior peripapillary quadrants [18]. This pattern of RGC damage may indicate a preferential contribution of parasol RGCs projecting to the magnocellular pathway (M-cells) [19,20], closely resembling that described in glaucoma. On the other hand, the axonal loss in Parkinson’s disease (PD) points toward the preferential involvement of the temporal quadrant of the optic disc, where the papillomacular bundle is located [21,22,23]. This pattern of axonal damage seems to resemble some mitochondrial optic neuropathies, such as Leber’s hereditary optic neuropathy, rather than glaucoma. This specific pattern of damage might be attributable to the parvocellular nature of the axons constituting the papillomacular bundle, which makes them more vulnerable to mitochondrial dysfunction and oxidative stress, probably due to the high energetic demand in relation to a low energetic potential [19].

### 1.3. Neurodegeneration and Microvascular Dysfunction

Neurodegenerative disorders represent the leading cause of disability worldwide, and their prevalence rises dramatically with age. As populations are aging in many countries, the health and social burden of these disorders is predicted to increase in the near future [24,25]. Although the clinical symptoms of neurodegenerative disorders occur late in the disease course, the underlying pathological alterations—such as amyloid accumulation and microvascular dysfunction—may develop subclinically years to decades before the disease onset [9,26,27].

Aging is associated with anatomical and functional changes in the cerebral vessels, which may compromise the neuronal function and increase the risk of neurodegeneration [28,29]. Although the etiology of AD is still unclear, accumulating evidence suggests that cerebral hypoperfusion may play a role in the onset and progression of the neurodegenerative process [30,31,32,33,34]. In particular, brain hypoxia and reduced glucose supply disrupt the blood–retinal barrier, thus inducing oxidative stress, inflammation, and dysregulation of nitric oxide. These events generate a vicious circle in which perfusion is further reduced, amyloid clearance is impaired, and neurodegeneration is accelerated [35].

In multiple sclerosis (MS), microvascular abnormalities and hypoxia might play a casual role in damage to oligodendrocytes and myelin resulting in the demyelinating lesions [36,37]. This is also supported by perfusion-weighted magnetic resonance imaging (MRI) studies that documented an impairment of cerebral blood flow in the white matter of patients with MS, regardless to the clinical subtype, which does not seem to be secondary to axonal degeneration with reduced metabolic demands [38,39,40].

Neurodegeneration occurs also in various ophthalmological conditions. Among these, glaucoma is a progressive optic neuropathy characterized by loss of retinal ganglion cells, damage to the visual field, and vision decline [41,42]. Additional mechanisms other than increased intraocular pressure (IOP) are involved in the neurodegeneration, which progresses despite a well-controlled IOP in a significant proportion of patients [43]. Reduced optic nerve perfusion and vascular dysregulation are suggested to be crucial factors in the development and progression of glaucoma, particularly in patients with normal IOP (i.e., normal tension glaucoma) [44,45].

Recent advances in retinal imaging have enabled improved visualization of the retinal vascularity. In particular, optical coherence tomography angiography (OCT-A) is a novel non-invasive imaging technique providing depth-resolved volumetric information about the choroidal and retinal microcirculation. This review aims at discussing recent findings on the application of OCT-A as a non-invasive tool for studying optic nerve alterations occurring in neurodegenerative disorders with a specific focus on the comparison with those detected in glaucoma.

## 2. Technical Principles of OCT-A

Optical coherence tomography angiography is a rapidly emerging imaging technique that is able to non-invasively produce angiographic images of the eye. The latter characteristic is obtained by detecting the motion of blood within vessels as a natural contrast agent.

Several studies have employed OCT-A to characterize the retinal and choroidal vessels. The retinal vessels are located in three retinal capillary beds: the superficial (SCP), middle (MCP), and deep capillary plexuses (DCP) [46,47]. In addition, a fourth vascular layer—the radial peripapillary capillary plexus (RPCP)—is located within the superficial RNFL surrounding the optic disc. Finally, OCT-A is effectively capable to study the innermost portion of the choroid that is termed choriocapillaris.

Volumetric structural OCT images are obtained by performing serial single B-scans at distinct retinal locations. Oppositely, OCT-A devices perform several repeated B-scans at the same retinal location. The interscan time (the delay between two repeated B-scans) has a crucial role in OCT-A imaging, as a shorter interscan time is associated with a reduction in sensitivity to motion, as well as a contraction in the occurrence of parasitic eye motions [48,49]. Importantly, the interscan time and the background noise determine the slowest detectable flow [49]. OCT-A technologies may use three different methodologies to detect flow: (i) phase-based; (ii) amplitude-based; and (iii) complex amplitude-based, in which OCT-A algorithm uses both phase and amplitude information.

Optical coherence tomography angiography images are mainly displayed with a two-dimensional (2D) en face visualization. Using the segmentation of the volumetric OCT-A scans at specific depths, 2D en face images are obtained by summing or projecting flow data within any slab (e.g., SCP). Recently, a three-dimensional (3D) visualization has proven to be beneficial in different macular affections, including macular edema due to retinal vascular disturbances [50], type 3 macular neovascularization associated with age-related macular degeneration [51], and diabetic retinopathy [52,53].

Artifacts represent a main problem in OCT-A imaging. Segmentation errors may affect 2D en face OCT-A images, which are visualized by placing two boundaries throughout the retinal and/or choroidal structure and associated with flattening of data in any given segmented volume. However, parameters used to differentiate retinal and choroidal layers may be significantly altered in pathological conditions and these boundaries may be thus placed in wrong positions [54]. Projection artifacts (decorrelation tails) may affect the visualization of the deeper vascular layers (ICP, DCP, and choriocapillaris). In order to visualize the deeper vessels, the OCT beam passes through the retinal layers, and vessels included in these layers may be falsely displayed on the en face OCT-A images of the deeper layers. In order to limit projection artifacts, previous papers employed the strategy to subtract the SCP OCT-A en face image from the deeper layers [55,56]. Shadowing artifacts occur when the OCT beam is attenuated or blocked thereby impeding its passage to the deeper layers of the retina/choroid. Different structures, including vitreous opacities and drusen may lead to this kind of artifact [54]. Finally, OCTA has still limited performance in the correct visualization of larger-sized choroidal vessels. This limitation is caused by the signal attenuation due to scattering by the retinal pigment epithelium and choriocapillaris [57]. Consequently, choroidal vessels are typically visualized as black silhouettes with complete loss of signal.

## 3. Alzheimer’s Disease

Alzheimer’s disease is the most common form of neurodegeneration of elderly people in western countries, with more than 15 million affected people worldwide [58]. This form of dementia is mainly due to an accumulation of debris, namely neurofibrillary tangles, that causes a neuronal loss passing through inflammation, oxidative stress, and vascular abnormalities [59,60]. Many factors are implicated in the pathogenesis of the AD, but the accumulation of the misfolded amyloid β-protein (Aβ) and hyperphosphorylation of tau proteins are considered the crucial hallmarks of the disease [61]. Patients with AD could be affected by different kinds of visual impairment, including deficits in contrast sensitivity and dyschromatopsia, deficits of the visual field, and alteration in visual evoked potentials [62]. Histopathological studies demonstrated extensive loss of retinal ganglion cells and their axons in the retinas from subjects with AD [63,64]. Furthermore, there is robust evidence from structural OCT studies of significant thinning of ganglion cell-inner plexiform layer, ganglion cell complex, macular volume, choroidal thickness, and peripapillary retinal nerve fiber layer thickness in patients with AD [65].

Based on the similarities between cerebral and retinal small vessels, several authors investigated the retinal microvasculature changes in patients with AD by using OCT-A (Table 1) [66,67,68,69,70,71,72,73,74,75,76]. In patients with clinical AD, OCT-A disclosed a reduced perfusion density of capillaries of both SCP [66,67,68,69,73] and DCP [68,75]. Furthermore, patients with AD showed a larger foveal avascular zone (FAZ) in comparison to healthy subjects [66,67,75]. Interestingly, Yoon et al. reported that the impairment of the SCP vascular density was associated with the enlargement of the infero-lateral ventricle on volumetric MRI [74]. To explain the retinal microvasculature abnormalities in AD, authors hypothesized different mechanisms, such as Aβ accumulation within the retinal capillaries, amyloid angiopathy, and binding of vascular endothelial growth factor to Aβ and its confinement in the plaque [66,70,73].

Since AD is usually diagnosed in the stage of dementia, when advanced neurodegeneration and vascular damage have already occurred, there is great interest in new biomarkers for the early diagnosis of the disease [77]. Several groups tried to elucidate the presence of OCT-A alterations in patients in the preclinical stage of AD, including the phase of mild cognitive impairment (MCI) [68,70,71,72,74]. Jiang et al. reported that patients with MCI had a lower vessel density of the DCP in the superior nasal quadrant compared to controls [68]. Another study documented an impairment of the SCP in patients with early AD and amnestic type MCI [76]. In addition, an enlargement of the FAZ was reported in subjects affected by preclinical AD diagnosed by positron emission tomography and/or cerebrospinal fluid testing [70]. Using another retinal technique, namely dynamic vessel analyzer, Querques et al. reported that the arterial dilation of the retina was decreased in both AD and MCI patients, and that this vessel response was correlated with the Aβ level in the cerebrospinal fluid [71]. However, these results were not confirmed by other authors, which found no differences in vessel density between MCI and control subjects [71,73], or even a higher density in subjects with preclinical AD [72]. For this reason, even if the data are promising, to date we are not able to conclude that OCT-A could identify patients affected by the preclinical stage of AD or MCI.

Some authors have investigated the changes in the peripapillary vasculature by means of optic nerve head OCT-A [69,75,76]. Lahme et al. reported a reduction of the peripapillary vessel density in patients with clinical AD [69]. Conversely, Zabel et al. found no significant differences in the peripapillary vascularity between patients with AD and control subjects [75]. In agreement with this, Zhang et al. reported a normal peripapillary vascularity in patients with early AD and amnestic type MCI [76]. Therefore, there is no conclusive evidence supporting the association between AD and impaired peripapillary vascularity.

In summary, OCT-A is able to detect microvascular retinal alterations of the macula that could be useful in the diagnosis and follow-up of patients affected by AD. Further studies are needed to confirm the promising data in the prodromal stages of the disease, in order to obtain a possible non-invasive and fast biomarker to identify individuals with early AD who are more likely to benefit from treatment.

## 4. Parkinson’s Disease

Parkinson’s disease is the second most common neurodegenerative disorder of the elderly population [78]. The disease is mainly caused by intracellular deposition of fibrillar α-synuclein, namely Lewy bodies, that leads to a progressive loss of dopaminergic neurons in the substantia nigra and in other sub-cortical nuclei [79]. The most common clinical features are different movement alterations such as bradykinesia, resting tremor, or rigidity and an irreversible deterioration in cognitive function. Moreover, visual symptoms—such as altered visual acuity, impaired contrast sensitivity, and worsened color vision—have been also reported [80]. Since the early description of ocular findings, different structural changes of the retina have been showed using SD-OCT. In particular, PD patients showed a significant thinning of inner retinal layer and retinal nerve fiber layer, suggesting that these parameters could be considered as surrogate markers for brain damage [81,82].

In addition to neurodegeneration, the vascular impairment has been identified as a possible key factor to the occurrence and progression of PD [83]. Recently, retinal microvessel status has been evaluated in patients with PD using OCTA [84,85]. Patients with early stage PD showed a reduced perfusion density of SCP in all the quadrants evaluated individually and in the total annular zone, defined as an area of 2.5 mm diameter centered on the fovea excluding the FAZ [84,85]. Given previous studies on animal models of PD reporting the accumulation of α-synuclein at the level of the wall of arteries, authors hypothesized that the same pathogenetic mechanism could explain the decreased perfusion density in SCP in PD patients [83]. Furthermore, in PD patients GCIPL thickness was positively correlated with the total annular zone of the SCP, supporting the hypothesis that microvessel impairment may contribute to the neurodegenerative process [84].

Shi and collaborators investigated also capillary complexity using fractal dimension analysis of OCTA images [85]. Interestingly, authors reported a reduced complexity in both the SCP and DCP. In addition, complexity in the SCP was negatively correlated with the thickness of the ganglion cell and inner plexiform layer and complexity of the DCP was negatively correlated with duration of the disease. This study suggests that complexity analysis of retinal capillary vasculature is able to detect subtle changes of vascular alteration in PD patients and that it shows a closer relationship with retinal structure [85].

In summary, retinal capillary impairment seems to occur early in the PD cascade. These findings suggest that OCTA could represent a new path for the investigation of PD and will likely be useful in the future as a valuable biomarker for the early detection of disease and progression over time.

## 5. Multiple Sclerosis

Multiple sclerosis (MS) is the most common chronic inflammatory disorder of the central nervous system, affecting more than 2 million people worldwide. The disease is thought to be due an autoimmune demyelinating process in which neuroaxonal degeneration and gliosis are the principal driver of disability [86]. Common clinical manifestations include muscle weakness, sensory deficits, cognitive impairment, and fatigue [86]. Moreover, ocular involvement is a common feature of MS. In particular, in approximately 25% of patients, optic neuritis (ON) represents the first manifestation of the disease in approximately 25% of patients, and an episode of 50% to 80% experience ON is experienced by 50–80% of patients during the disease course [87].

Recent studies aimed to identify retinal biomarkers in order to facilitate the diagnosis and monitoring of disease activity, and OCT-derived parameters emerged for this purpose. Interestingly, OCT disclosed both peripapillary and macular RNFL thinning in patients with MS. Particularly, both patients with a history of optic neuritis (MSON) and those without (MSNON) presented a significant retinal impairment [88]. In addition to demyelization, vascular dysfunction has been described in MS. Indeed, endothelial dysfunction probably secondary to inflammation and a chronic state of impaired venous drainage from the central nervous system, seem to play an important role in the development and course of the disease [89].

There is emerging evidence that OCTA could be considered as an effective tool in detecting pathological alterations occurring in the retinal vasculature in patients with MS. The optic nerve head flow index, representing the optic nerve head blood flow velocity, measured using OCTA, has been reported to be significantly reduced in patients with MS compared with healthy controls, particularly in those with a history of ON [90,91]. In addition, recent studies evaluated macular vascular density in patients with MS using OCTA. However, no clear trend toward variations of vascular density has been identified. Indeed, in recent reports, vessel density of the SCP and DCP showed variations in both directions (i.e., increased or decreased) (Table 2) [92,93,94,95,96,97,98,99]. Some authors reported a decreased SCP in both patients with MSON and MSNON [92,95,97,98]. Conversely, Feucht et al. found a decreased SCP and DCP only in eyes with a history of ON. Thus, authors could not detect any signs of ON-independent impairment of retinal vascularization during MS [96]. Authors hypothesized that neuronal and axonal decline result in reduced metabolic activity within the inner retinal layers with consecutive lower oxygen and blood demand and regression of vessels of the SCP. Since the DCP is supplied by anastomoses from superficial vessels, this could secondarily also affect deeper retinal vessel structures [96].

However, these studies did not take into account the structural retinal changes that occur in patients with MS, and in particular, the reduction of RNFL and GCIPL. Jiang et al. evaluated the volumetric vessel density in patients with relapsing-remitting MS, considering also retinal volume changes in order to better estimate vessel density. Surprisingly the study revealed a significant increasing of volumetric vessel density in both MSNON and MSON patients. In addition, volumetric vessel density was positively correlated to the Expanded Disability Status Scale (EDDS) and disease duration, and negatively related to visual function. Authors hypothesized that increased vascular density could be the result of diffuse chronic inflammation and related hypoxia-induced neoangiogenesis [99].

Lanzillo et al., evaluated longitudinally patients with stable MS after 1-year follow up, reporting a significant increase in the parafoveal vascular density. Since all patients were on a stable disease modifying treatment and did not present any recent relapses of the disease, authors speculated that the increase of VD over time could a consequence of treatment. Moreover, parafoveal vascular density was negatively correlated with the EDDS, which is one of the most common used score to assess disability due to MS [93]. According to these results, Murphy et al., showed that SVD was negatively correlated with both EDDS and multiple sclerosis functional composite scale [98]. These results highlight the role of retinal vascular density as a possible novel biomarker to monitor MS progression.

It has been shown that OCTA examination in patients with MS are more likely to present images artifacts that lead to underestimate vessel density measurements. This issues should be considered in the evaluation of OCTA images of MS patients and a subjective assessment of presence and severity of different artifact is advisable [54].

In summary, OCTA reveals an impairment of retinal vascularization in patients with MS and shows a promising role to detect early microvascular alterations, but also as a potential novel biomarker to monitor the disease progression.

## 6. Optic Nerve Degenerative Diseases

The ophthalmoscopic examination of the optic disc may not be sufficient to differentiate the various optic neuropathies, since the majority of them are characterized by either optic disc edema or optic atrophy. Optical coherence tomography angiography has been proposed as a useful non-invasive tool to gain a better visualization of the optic nerve head vasculature [100]. Falavarjani et al., assessed optic disc vasculature with swept-source OCT-A in different optic neuropathies, namely non-arteritic anterior ischemic optic neuropathy (NA-AION), Leber’s hereditary optic neuropathy, autosomal dominant optic atrophy, autoimmune optic neuritis, and idiopathic intracranial hypertension. All types of optic neuropathies were associated with a reduction in peripapillary vascular blood flow, which was more substantial in eyes with optic atrophy [100]. It is still not clear if the reduction of peripapillary vascular blood flow is consequent to the damage to the optic disc microvasculature, the reduced metabolic request due to the smaller number of fibers, or a combination of both mechanisms.

The differential diagnosis between with arteritic anterior ischemic optic neuropathy (A-AION) and NA-AION has important clinical implications because the former may lead to profound bilateral loss of vision that may be prevented with high-dose corticosteroid treatment [101]. In patients with NA-AION, OCT-A studies revealed perfusion deficit at the level of radial peripapillary capillaries with preserved choriocapillaris perfusion [102,103,104]. On the other hand, two studies evaluating the vascular changes in patients with A-AION reported choriocapillaris perfusion deficit on OCT-A [105,106]. Choroidal hypoperfusion is considered a distinctive feature of A-AION, in which inflammation and occlusion of posterior ciliary arteries occurs proximally to their division into paraoptic and choroidal branches [107]. Therefore, OCT-A may have a role in distinguish A-AION from NA-AION.

Leber’s hereditary optic neuropathy is a mitochondrial neurodegenerative disease affecting retinal ganglion cells and their axons in the optic nerve characterized by bilateral subacute loss of central vision during young adult life [108]. The disease is associated with distinctive abnormalities of peripapillary blood vessels including telangiectatic microangiopathy and small vessel tortuosity [109]. A prospective multicenter study reported a reduction in vascular blood flow in the temporal sector of the optic nerve in the subacute phase of Leber’s hereditary optic neuropathy, and a reduction in all sectors in the chronic phase [110]. Borrelli et al. showed that eyes with Leber’s hereditary optic neuropathy present SCP and DCP changes that are mainly confined to the nasal and inferior parafoveal sectors corresponding to the papillomacular bundle. In addition, visual loss was associated with SCP flow impairment, but not with OCT-detectable structural damage [111]. Microvascular impairment in the macular and peripapillary regions were also demonstrated in autosomal dominant optic atrophy, which represents the most common hereditary optic neuropathy [112].

Cennamo et al., evaluated the morphological and vascular changes in three congenital optic abnormalities, namely morning glory papilla, optic disc coloboma, and optic pit. In eyes with morning glory papilla, OCT-A revealed a dense peripapillary microvascular network, which instead was absent in both coloboma and optic pit. These features may contribute to the understanding of the pathogenesis of these three congenital conditions rather than help to the differential diagnosis, which is mostly reached by fundoscopy examination [113].

Radiation optic neuropathy is a late complication of ocular irradiation resulting in severe and irreversible vision loss. The condition is thought to be caused by vascular endothelial injury resulting in thrombosis and neuronal ischemia [114]. Parrozzani et al. proposed a 0–4 grading system of radiation optic neuropathy based on OCT-A findings: grade 0 = normal peripapillary capillaries; grade 1 = initial loss of radial peripapillary capillary plexus; grade 2 = peripapillary hypoperfusion in less than 2 quadrants; grade 3 = peripapillary hypoperfusion in more than 2 quadrants; grade 4 = diffuse peripapillary hypoperfusion. The above mentioned grading system well correlated with visual acuity of the affected eyes [115].

## 7. Glaucoma

Since its introduction in clinical practice, OCT-A has helped clinicians and scientists to evaluate the relationship existing between neuronal and vascular components in glaucomatous eyes. Initially, the attention was focused on the peripapillary microvasculature and, more specifically, on the RPCP vessel density. Capillary dropouts in areas corresponding to peripapillary RNFL defects were demonstrated [116,117,118,119,120].

More recently, macular microvasculature was also investigated in glaucoma, and several studies reported significantly decreased SCP vessel density, with relative sparing of the DCP [121,122,123,124]. The SVC has been found to supply blood to the nerve fiber layer and ganglion cell layer. Thus, these studies suggested that vascular changes associated with glaucoma occurred preferentially among the vessels that feed the superficial layers of the retina. Takusagawa et al. noticed that a macular area with low perfusion in the SVC corresponded in shape, size, and location to areas of detectable GCC thinning and VF defects [121]. This indicates that there is an intimate correspondence between vascular and structural defects. It still needs to be clarified whether the optic disc or macular vascular changes are a cause or a consequence of the glaucomatous loss of RGCs and their axons. The correspondence of shape and location between vascular abnormalities detected on OCT-A and macular GCIPL and peripapillary RNFL defects may suggest that the vascular density is reduced because of the loss of neural tissue, and not the other way around.

## 8. Conclusions

Optical coherence tomography angiography allows non-invasive evaluation of the microvasculature in the retina, which represents the most accessible part of the central nervous system. A large body of evidence indicates that ocular neurodegenerative conditions such as glaucoma and central nervous system diseases such as AD, PD, and MS are characterized by retinal microvascular impairment. The pathological mechanisms underlying these vascular changes still need to be fully clarified, and further studies are required to define the casual relationship between microvascular impairment and neuronal damage.

Since neurodegenerative disorders are often diagnosed when extensive neuronal damage has already occurred, many efforts have been spent to identify useful biomarkers that can be used for the early diagnosis and monitoring of the degree of neural degeneration. Optical coherence tomography angiography may represent a quick, inexpensive, and non-invasive biomarker for neurodegenerative disorders. Although the clinical utility of OCT-A in the preclinical stages requires further confirmation, its sensitivity and specificity might be improved by integrating it in a multimodal imaging approach. Future research should explore the diagnostic and prognostic value of OCT-A combined with other biomarkers such as structural OCT measurements, brain MRI, cerebrospinal fluid testing, and amyloid positron emission tomography.

## Figures and Tables

**Table 1:** Characteristics of studies evaluating ocular coherence tomography angiography in patients with Alzheimer’s disease.

Author (Year)	Patients	MMSE	OCT-A Scan	Main Results
Bulut et al. (2018)	16 AD, 16 controls	16.92/26.81	Macula 6 × 6 mm	Reduced SCP vessel density and enlargement of the FAZ in patients with AD
Grewal et al. (2018)	2 discordant twins	NA	Macula 6 × 6 mm	Reduced SCP density and larger FAZ in the twin with AD
Jiang et al. (2018)	12 AD, 19 MCI, 21 controls	NA	Macula 3 × 3 mm	Reduced SCP and DCP vessel density in patients with AD; reduced DCP density in superior nasal quadrant in MCI.
Lahme et al. (2018)	36 AD, 38 controls	22.32/NA	Macula 3 × 3 mm, ONH 4.5 × 4.5 mm	Reduced SCP and peripapillary vessel density patients with AD
O’Bryhim et al. (2018)	14 preclinical AD, 16 controls	NA	NA	FAZ enlargement in patients with preclinical AD
Querques et al. (2019)	12 AD, 12 MCI, 32 controls	20.7/24.9/NA	Macula 3 × 3 mm and 6 × 6 mm	No differences in SCP, DCP, choriocapillaris, and choroid perfusion density
van de Kreeke et al. (2020)	13 preclinical AD, 11 controls (twins)	29.0	Macula 3 × 3 mm, ONH 6 × 6 mm	Increased macular and peripapillary vessel density in patients with preclinical AD; no difference in area of the FAZ
Yoon et al. (2019)	39 AD, 37 MCI, 133 controls	20.1/22.6/29.2	Macula 3 × 3 mm and 6 × 6 mm	Reduced SCP vessel density and perfusion density in patients with AD; no difference between MCI and controls
Yoon et al. (2019)	9 AD, 7 MCI	21.6/26.0	Macula 3 × 3 mm and 6 × 6 mm	Correlation of infero-lateral ventricle MRI volume with retinal vessel density (3 mm and 6 mm) and perfusion density (3 mm)
Zabel et al. (2019)	27 AD, 27 POAG, 27 controls	20.55/28.92/28.39	Macula 3 × 3 mm, ONH 4.5 × 4.5 mm	Reduced DCP vessel density and enlargement of the FAZ in patients with AD
Zhang et al. (2019)	16 early AD, 16 controls	20.25/27.06 *	Macula 3 × 3 mm, ONH 4.5 × 4.5 mm	Reduced SCP vessel density and adjusted flow index in patients with early AD

AD: Alzheimer’s disease; DCP: deep capillary plexus; FAZ: foveal avascular zone; MCI: minimal cognitive impairment; MMSE: mini mental state examination; NA: not available; ONH: optic nerve head; POAG: primary open angle glaucoma; SCP: superficial capillary plexus. * Montreal Cognitive Assessment.

**Table 2:** Characteristics of studies evaluating ocular coherence tomography angiography in patients with multiple sclerosis.

Author (Year)	Patients	OCT-A Scan	Main Results
Jiang et al. (2020)	MSNON = 123, MSON = 36, HC = 198	Macula 3 × 3	SVD increased in MSNON compared to HC; DVD decreased in MSON compared to MSNON and HC; VVDs * increased in MSON; VVDd * increased in MSNON and MSON
Ulusoy et al. (2020)	MSNON + MSON = 20, HC = 24	NA	SVD reduced in MSNON/MSON compared with HC
Feucht et al. (2019)	MSNON = 25, MSON = 17, HC = 50	Macula 6 × 6	SVD NS between MSNON compared to HC, decrease in MSON; DVD NS between MSNON compared to HC, decrease in MSON
Murphy et al. (2019)	MSNON + MSON = 111, HC = 50	Macula 3 × 3	SVD decreased in MSON and MSNON compared to HC, decreased in MSON compared to MSNON, DVD NS between MSNON/MSON and HC and between MSON and MSNON
Yilmaz et al. (2019)	MSNON + MSON = 47, HC = 61	Macula 3 × 3	SVD decreased in MSNON + MSON compared with HC, decreased in MSON compared with MSNON, DVD decreased in MSNON/MSON compared with HC
Lanzillo et al. (2019)	MSNON = 27, MSON = 23, HC = 46	Macula 6 × 6	SVD parafoveal increase in MSNON/MSON compared with HC after 1 year follow-up
Lanzillo et al. (2018)	MSNON = 27, MSON = 23, HC = 46	Macula 6 × 6	SVD decreased parafoveal SVD in MSNON/MSON compared to HC, increased foveal SVD in MSNON/MSON compared to HC
Spain et al. (2018)	MSNON + MSON = 45, HC = 32	ONH 3 × 3	ONH-FI decreased in MSNON/MSON
Wang et al. (2014)	MSNON = 25, MSON = 10, HC = 21	ONH 3 × 3	ONH-FI decreased in MSON

MSNON: multiple sclerosis without history of optic neuritis; MSON: multiple sclerosis with history of optic neuritis; HC: healthy control; ONH: optic nerve head; SVD: vessel density of the superficial vascular plexus; DVD: vessel density of the deep vascular plexus; VVDs: volumetric vessel density in the superficial vascular plexus; VVDd: volumetric vessel density in the deep vascular plexus; NS: not significant; ONH-FI: Optic Nerve Head Flow Index. * based on fractal dimension for VD and VVD.
